# Dyspepsia and Consumption

**Published:** 1896-03-21

**Authors:** Solomon C. Smith

**Affiliations:** Physician to the Westminster General Dispensary.


### Dyspepsia and Consumption.

By Solomon C. Smith, M.D., M.R.C.P., Physician to the Westminster General Dispensary.

### III.—TREATMENT.

From what has been already said it will have been gathered that, with the exception of what has been spoken of as the terminal dyspepsia, I do not regard the indigestions connected with consumption as in any way special in themselves. As might, then, be expected, there is but little to say in relation to treatment which differs from what might be said in describing the treatment of similar conditions met with in other relationships. Nevertheless, it will not be without interest to consider some of the salient points in the treatment of the conditions most frequently met with. The treatment of the dislike to fat is to a large extent a matter of careful search for some form of it which can be digested. If meat fat cannot be taken, it may be found that fresh butter, or bacon, or cream cheese, or sardines, and especially various forms of blancmange and stewed fruit with cream, can be digested without discomfort. Above all must the extraordinary fact be borne in mind that cod liver oil, the pure and naked article, will often be digested when a bit of fat on a mutton chop will cause the severest discomfort. It becomes, then, far more important to discover a substitute than to persevere in the use of anything that disagrees. From the rapidity with which the discomfort follows the ingestion of fat, it would appear that it undergoes some change almost as soon as it enters the stomach. It is a matter of idiosyncrasy, and certain fatty substances seem, as one might say, to act as poisons to the individual. This has a bearing upon the medicinal treatment of the condition. Food must not be allowed to lodge in the stomach, and it has always appeared to me that so far as drugs have been of service, those which have been of most use have been such as may be imagined to hasten the passage of the food from the stomach into the intestines. Of these, rhubarb, ipecacuanha, and strychnine have seemed to be the most effectual. They should be taken immediately before meals. It seems probable also that the indigestion of fat has some relation to an imperfect formation of gastric juice; at any rate, it is not uncommonly the case that after a little stimulant, or some clear soup, has been partaken of, fat can be digested which beforehand would have disagreed. In view of the considerable proportion of the cases of phthisis which are accompanied by dilatation of the stomach, and the tendency which exists in that condition to the accumulation of acrid ferments, and the dilution of the normal secretions of the stomach, it is well to caution patients against the bad habit of drinking quantities of fluids at meal times, by which the activity of the gastric juice is very much weakened.

For the anorexia which so often accompanies phthisis quinine is very useful, if it agrees, which, however, is not always the case; and, speaking empirically, I have found a mixture of hypophosphite of soda with spirit of chloroform or glycerine, combined with tincture of gentian or calumba, more serviceable than the stronger bitters.

Speaking of tonics makes one revert to the question of giving iron. This hardly comes into question during the more active stages of dyspepsia, but when the tongue is fairly clean and flabbiness and pallor are its chief peculiarities, and especially in the early or premonitory stages of the disease, iron may often be given with marked advantage, sometimes in the form of the phosphate syrup or the tincture of the perchloride, more often as the carbonate in the form of jelloids, or Blaud's pills, both of which may be combined with small doses of arsenic.

The treatment of the vomiting which is produced by cough, and the cough which is set up by food in the stomach, and too often goes on until vomiting is produced, have already been referred to. It is worth bearing in mind that to those who can vomit easily, the complete emptying of the stomach, the bronchial tubes, and probably to some extent the cavities also, which is produced by a morning vomit, is a considerable gain, for by one act a great deal of deleterious material is got rid of, part of which would have required much coughing to remove, and much of which would have had to pass through the intestines before it could be got rid of.

The fermentive dyspepsia which troubles so many phthisical patients is a great difficulty. Their debility makes one chary of using the stomach tube, while the purgatives which are so useful in cases in which this condition occurs in stronger people are here out of the question. Much benefit, however, is derived from restricting the amount of fluid drunk at meals, and from the administration of carbolic acid, creasote (which can now be obtained in minute capsules which are very easily taken), and sodium sulpho-carbolate. These should all be given along with food, without waiting for the fermentation to occur; charcoal, however, combined if necessary with the carbonates of soda and magnesia, may be administered when the signs of decomposition show themselves, and often with great advantage. Throughout the whole progress of consumption attacks of gastro-duodenal catarrh are apt to occur which are best relieved by the old familiar remedy of a blue pill, or a dose of grey powder, followed by a morning draught of compound senna mixture; and in regard to these intercurrent attacks it is to be observed that the depression produced by this line of treatment is far less injurious than is the innutrition produced by low diet prolonged over a longer space of time. In the more torpid forms of the precursory indigestion it is especially worth remembering that the occasional use of mercurials is advantageous as an alternative to the iron and other tonics which are so useful in that condition.

In the terminal dyspepsia, as described by Fenwick, recourse must be had to artificially peptonised foods. But the diarrhœa is often a great difficulty, generally requiring the administration of opium for its relief, and the weakness accompanying it frequently demands the pretty free administration of alcohol.

Finally, I would repeat that throughout the whole course of phthisis careful and painstaking attention to the digestive peculiarities of the individual will repay the practitioner more than almost anything else that he can do in the treatment of consumption.

